# Supplementary material for: Sequence structure in children’s speech reveals non-linear development of relations between word categories
Source: Commun Psychol. 2025 Dec 26;4:12. doi: 10.1038/s44271-025-00380-w (PMC12847997; doi:10.1038/s44271-025-00380-w)
Supplement: Supplementary file 2 — Supplementary Information [file 44271_2025_380_MOESM2_ESM.pdf]

Supplementary Information: Sequence structure in children's speech reveals non-linear development of relations between word categories

Maja Linke[1*] and Michael Ramscar[2]

[1*]Max Planck Institute for Human Cognitive and Brain Sciences, Stephanstr. 1A, Leipzig, 04103, Germany.
[2]Department of Psychology, University of Tuebingen, Schleichstr. 4, Tuebingen, 72074, Germany.

*Corresponding author(s). E-mail(s): linke.maja@gmail.com;

1 Supplementary Materials

1.1 Words used in the reported study

TIME

time, number, day, morning, week, night, last, days, tomorrow, bedtime, while, once, nighttime, during, sunday, moment, saturday, summer, until, story, after, question, company, year, before, thursday, till, minute, every, tonight, second, ago, midnight, minutes, later, magic, color

NAMES

daniel, sarah, michael, john, tommy, jessica, sara, johnny, andy, emily, robert, katie, michelle, thomas, samantha, bob, amanda, lad, aunt, sally, toby, julie, bill, christopher, danny, bobby, jack, tom, megan, danielle, joe, rachel, jessie, alex, jonathan, trevor, mary, maria, mike, lucy, paul, jennifer, joey, adam, tim, ron, james, joshua

FOOD

juice, cakes, cracker, lemonade, turkey, tea, carrots, cake, cereal, milk, chips, carrot, flour, meat, tasting, chip, raspberry, angel, cookie, syrup, sandwiches, corn, rice, fan, bullfrog, pumpkin, comb, necklace, fruit, beans, yogurt, tap, oatmeal, pudding, coffee, cocoa, koolaid, macaroni, drinks, magazine, cane, potatoes, applesauce, broom, banana, elf, breakfast, tissue, pig, ham

VERBS

be, do, go, get, want, have, put, see, come, make, know, eat, look, take, say, play, need, sit, like, find, read, think, build, wash, drive, sing, cough, choose, grab, glue, pinch

FAMILY

father, mother, sister, brother, aunt, uncle, cousin, grandmother, grandfather

PRONOUNS

she, her, hers, they, them, theirs, he, him, his

NUMBERS

one, two, three, four, five, six, seven, eight, nine, ten, eleven

COLORS

yellow, blue, green, red, white, black, pink, purple, orange, gray, brown, grey

List of CHILDES Corpus IDs

1, 14, 16, 19, 26, 38, 46, 48, 49, 51, 52, 60, 62, 63, 66, 70, 74, 77, 222, 225, 229, 262, 263, 264, 265, 268, 269, 271, 272, 274, 273, 275, 276, 277, 283, 284, 286, 287, 303, 312, 317, 325, 338, 339, 375, 376, 379, 381, 382, 223, 224, 227, 232, 233, 261, 267, 270, 278, 279, 280, 281, 282, 294, 305, 322, 332, 334, 335, 336, 337, 340, 386, 387, 75, 78, 79, 80, 218, 219, 266, 315, 380

1.2 Analyses of permutation entropy

To evaluate whether word order reflects local, sequence-level fluctuations and to test whether short conversational segments exhibit ordering patterns beyond those dictated by grammar, we calculated normalized permutation entropy [PE; 1] for each utterance ($m = 4$, $\tau = 1$). PE ranges from 0 (fully predictable) to 1 (where all $m!$ possible orderings are equally likely). In our age-stratified samples, mean PE values were high across all cohorts, approaching ~ 0.98 once utterances exceeded three words (Figure S9), with words categorized by frequency rank and part-of-speech. This finding aligns with previous reports of geometric distributions in conversational speech [2]. When word or part-of-speech distributions approximate a geometric (memoryless) form, local orderings become nearly random, and permutation entropy approaches its theoretical maximum—indicating an absence of sequential constraints beyond the next word. These combined metrics suggest that, at this scale, local sequences behave almost like random draws. We revisit this point in Section 3.5.

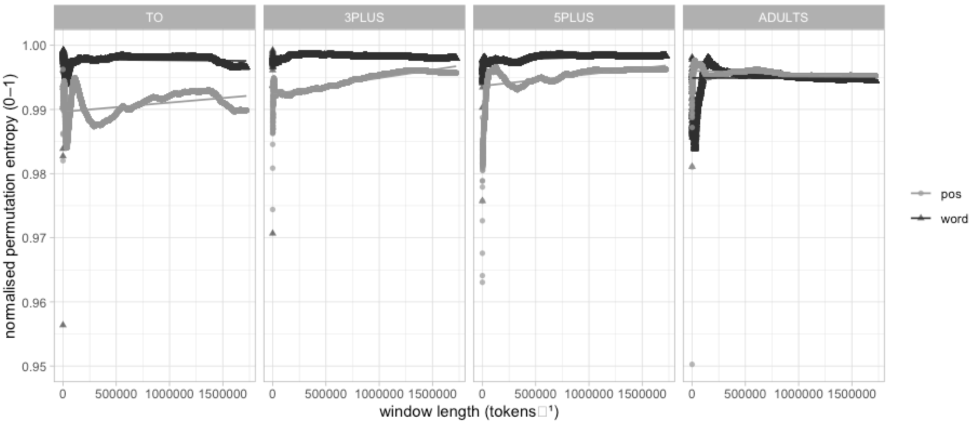

Supplementary Figure S1 Normalised permutation entropy (PE) as a function of window length, computed over sequences of part-of-speech tags (light grey) and frequency-ranked word tokens (black) for four speaker groups (TO: toddlers; 3PLUS; 5PLUS; ADULTS). Each point reflects PE over a fixed-length, non-overlapping window (order $m = 4$, delay $\tau = 1$). PE rises rapidly with window length and plateaus near 1.0 across groups and encoding types, indicating that local sequential order becomes increasingly indistinguishable from random permutations as speech aggregates grow. The faster saturation of word-level PE compared to part-of-speech PE reflects the higher diversity and flatter distribution of surface word forms.

Supplementary Table S1 Corpus Statistics, by cohort summary

	Utterances				Words			
Cohort	n_{utt}	mn_{utt}	sd_{utt}	md_{utt}	n_{wds}	mn_{wds}	sd_{wds}	md_{wds}
TODDLER	715086	2.267	1.829	2	1693641	3.588	1.731	3
3PLUS	539672	3.066	2.708	2	1750007	3.529	1.701	3
5PLUS	350963	4.737	4.186	4	1721828	3.636	1.752	3
ADULTS	246905	5.533	4.135	5	1742885	3.874	2.123	3

Supplementary Table S2 Mantel test results for diagonal (within–category) similarity across cohorts. Entries are Mantel r values (Pearson correlations between distance matrices) with significance based on 9,999 permutations.

	Euclidean			Mahalanobis		
Category	TO–3P	3P–5P	5P–AD	TO–3P	3P–5P	5P–AD
ALL	0.557***	0.618***	0.509***	0.371***	0.323***	0.228***
PRONOUNS	0.884***	0.854***	0.710***	0.797***	0.867***	0.733***
NUMBER	0.916***	0.890***	0.616*	0.866***	0.828***	0.570*
COLOR	**0.387**	**0.269**	**-0.023**	0.444*	**0.247**	**0.011**
NAME	**0.035**	**0.142**	**0.042**	**0.113**	**0.150**	**0.047**
FAMILY	0.711***	0.492*	**-0.181**	**0.411**	**0.287**	**-0.178**
VERB	0.718***	0.765***	0.581***	0.622***	0.634***	0.531***
FOOD	0.562***	0.427***	0.259***	0.450***	0.377***	0.218**
TIME	0.476***	0.580***	0.611***	0.438***	0.411***	0.546***

Supplementary Table S3 Correlation coefficients (Pearson's r) with permutation-based p-values (9999 permutations). Cells show correlations within and across lexical categories for each pair of cohort-specific embedding spaces. Bold values indicate estimates not meeting the $\alpha = .05$ threshold after permutation testing.

TODDLER – 3PLUS

Category	TIME	FOOD	VERB	KIN	NAME	COLOR	NUM	PRON
TIME	0.47***	0.42***	0.43***	**0.28**	0.13***	0.80***	0.36***	**0.30**
FOOD	0.41***	0.55***	0.57***	**0.31**	0.08**	0.41**	**0.26**	**-0.01**
VERB	0.42***	0.53***	0.70***	0.67***	0.28***	0.44***	**0.05**	0.84***
FAMILY	0.27***	0.38***	0.52***	0.66***	**0.05**	**0.27**	0.52***	0.62***
NAME	0.11***	0.19***	0.30***	**-0.09**	**0.03**	**0.08**	-0.35*	0.55***
COLOR	0.32***	0.35***	0.52***	0.34*	**0.04**	**0.24**	**0.25**	**0.37**
NUMBER	0.26***	0.38***	0.27***	0.73***	0.26***	0.84***	0.91***	0.80***
PRON	0.38***	0.36***	0.79***	0.67***	0.14**	**0.16**	**0.38**	0.90***

3PLUS – 5PLUS

Category	TIME	FOOD	VERB	KIN	NAME	COLOR	NUM	PRON
TIME	0.55***	0.27***	0.50***	0.63***	0.16***	0.85***	0.86***	0.65***
FOOD	0.18***	0.43***	0.59***	**0.09**	0.21***	0.39*	**0.20**	**0.17**
VERB	0.36***	0.39***	0.77***	0.61***	0.25***	0.54***	**0.25**	0.88***
FAMILY	**0.07**	0.25***	0.42***	0.41**	**0.09**	**-0.16**	-0.31*	**0.07**
NAME	0.10**	0.31***	0.17***	**0.15**	0.14***	**-0.14**	0.48***	**0.13**
COLOR	0.31***	0.39***	0.51***	0.61***	**-0.10**	0.32*	0.37**	0.65***
NUMBER	0.45***	0.21***	0.43***	0.62***	0.21***	0.68***	0.87***	0.88***
PRON	0.36***	0.23***	0.63***	0.40*	0.15**	0.40*	0.88***	0.86***

5PLUS – ADULTS

Category	TIME	FOOD	VERB	KIN	NAME	COLOR	NUM	PRON
TIME	0.58***	0.17***	**0.07**	**0.17**	**0.04**	0.81***	0.68***	**0.33**
FOOD	0.23***	0.25***	0.41***	**0.30**	**-0.03**	**0.26**	**-0.16**	**0.36**
VERB	0.32***	0.35***	0.57***	0.46**	0.21***	**0.19**	**0.22**	0.56***
FAMILY	0.30***	**-0.06**	0.20***	**-0.19**	**0.09**	**-0.21**	**-0.10**	**-0.11**
NAME	0.22***	**0.03**	0.16***	**0.12**	**0.02**	**-0.19**	-0.44***	0.39*
COLOR	0.18***	0.10*	0.31***	**0.16**	**-0.02**	**-0.06**	0.28*	**0.22**
NUMBER	0.29***	**0.05**	**0.01**	0.52***	**0.06**	**-0.23**	0.49***	0.37*
PRON	0.38***	0.17***	0.27***	0.36**	0.31***	**-0.08**	0.35**	0.69***

Supplementary Table S4 Parametric coefficients from the GAMM predicting lexical diversity. The table reports estimates, standard errors, t-values, two-sided p-values, and 95% confidence intervals computed as $\beta \pm 1.96 \times \text{SE}$. Confidence intervals are provided for transparency and comparability across models, although interpretation focuses on modelled smooths (reported separately). Significant effects at $\alpha = .05$ are shown in **bold**.

**Term**	**Estimate**	**SE**	**95% CI**	t	p
**(Intercept)**	**4.103**	0.105	**[3.897, 4.309]**	39.002	< .0001
**3PLUS**	**0.728**	0.149	**[0.435, 1.021]**	4.896	< .0001
**5PLUS**	**1.284**	0.149	**[0.992, 1.576]**	8.633	< .0001
**ADULTS**	**1.915**	0.149	**[1.623, 2.207]**	12.871	< .0001
**ADP**	**-0.912**	0.149	**[-1.205, -0.619]**	-6.131	< .0001
**ADV**	**-0.369**	0.149	**[-0.661, -0.077]**	-2.480	.013
**AUX**	**-1.033**	0.149	**[-1.326, -0.740]**	-6.940	< .0001
**CCONJ**	**-2.814**	0.149	**[-3.102, -2.526]**	-18.919	< .0001
**DET**	**-1.752**	0.149	**[-2.044, -1.460]**	-15.297	< .0001
**INTJ**	**-1.803**	0.150	**[-2.101, -1.505]**	-11.990	< .0001
**NOUN**	**1.793**	0.149	**[1.501, 2.085]**	12.051	< .0001
**NUM**	**-1.662**	0.152	**[-1.964, -1.360]**	-10.916	< .0001
**PART**	**-2.441**	0.149	**[-2.733, -2.149]**	-16.410	< .0001
**PRON**	**-0.552**	0.149	**[-0.844, -0.260]**	-3.714	< .001
**PROPN**	**0.560**	0.149	**[0.268, 0.852]**	3.766	< .001
**SCONJ**	**-2.025**	0.150	**[-2.324, -1.726]**	-13.462	< .0001
**VERB**	**0.948**	0.149	**[0.656, 1.240]**	6.370	< .0001
3PLUS:ADP	-0.332	0.210	[-0.744, 0.080]	-1.576	.115
**5PLUS:ADP**	**-0.585**	0.210	**[-0.997, -0.173]**	-2.781	.005
**ADULTS:ADP**	**-1.247**	0.210	**[-1.659, -0.835]**	-5.926	< .0001
3PLUS:ADV	-0.060	0.210	[-0.472, 0.352]	-0.286	.775
5PLUS:ADV	-0.178	0.210	[-0.590, 0.234]	-0.848	.400
**ADULTS:ADV**	**-0.558**	0.210	**[-0.970, -0.146]**	-2.653	.010
3PLUS:AUX	-0.292	0.210	[-0.704, 0.120]	-1.387	.166
**5PLUS:AUX**	**-0.582**	0.210	**[-0.994, -0.170]**	-2.769	.006
**ADULTS:AUX**	**-1.260**	0.210	**[-1.672, -0.848]**	-5.990	< .0001
**3PLUS:CCONJ**	-0.549	0.210	**[-0.961, -0.137]**	-2.610	.009
**5PLUS:CCONJ**	-0.836	0.210	**[-1.248, -0.424]**	-3.975	< .0001
**ADULTS:CCONJ**	-1.169	0.210	**[-1.581, -0.757]**	-5.556	< .0001
3PLUS:DET	-0.391	0.210	[-0.803, 0.021]	-1.860	.063
**5PLUS:DET**	**-0.692**	0.210	**[-1.104, -0.280]**	-3.287	.001
**ADULTS:DET**	**-1.280**	0.210	**[-1.692, -0.868]**	-6.085	< .0001
3PLUS:INTJ	-0.140	0.212	[-0.555, 0.275]	-0.663	.507
5PLUS:INTJ	-0.197	0.212	[-0.612, 0.218]	-0.932	.352
**ADULTS:INTJ**	**-1.303**	0.212	**[-1.718, -0.888]**	-8.085	< .0001
3PLUS:NOUN	-0.091	0.210	[-0.503, 0.321]	-0.434	.664
5PLUS:NOUN	-0.208	0.210	[-0.620, 0.204]	-0.988	.323
**ADULTS:NOUN**	**-0.444**	0.210	**[-0.856, -0.032]**	-2.587	.0098
3PLUS:NUM	-0.186	0.213	[-0.604, 0.232]	-0.873	.383
5PLUS:NUM	-0.400	0.213	[-0.818, 0.018]	-1.879	.060
ADULTS:NUM	-0.280	0.213	[-0.698, 0.138]	-1.316	.188
**3PLUS:PART**	-0.515	0.210	**[-0.927, -0.103]**	-2.448	.014
**5PLUS:PART**	-0.883	0.210	**[-1.295, -0.471]**	-4.197	< .0001
**ADULTS:PART**	-1.631	0.210	**[-2.043, -1.219]**	-7.753	< .0001
3PLUS:PRON	-0.375	0.210	[-0.787, 0.037]	-1.781	.075
**5PLUS:PRON**	-0.725	0.210	**[-1.137, -0.313]**	-3.445	< .001
**ADULTS:PRON**	-1.370	0.210	**[-1.782, -0.958]**	-6.510	< .0001
3PLUS:PROPN	-0.278	0.210	[-0.690, 0.134]	-1.322	.186
**5PLUS:PROPN**	-0.437	0.210	**[-0.849, -0.025]**	-2.079	.038
**ADULTS:PROPN**	-0.761	0.210	**[-1.173, -0.349]**	-3.618	< .001
3PLUS:SCONJ	-0.213	0.212	[-0.629, 0.203]	-1.006	.315
5PLUS:SCONJ	-0.281	0.212	[-0.697, 0.135]	-1.330	.184
**ADULTS:SCONJ**	-0.800	0.212	**[-1.216, -0.384]**	-3.782	< .001
3PLUS:VERB	0.010	0.210	[-0.402, 0.422]	0.047	.963
5PLUS:VERB	0.072	0.210	[-0.340, 0.484]	0.342	.733
ADULTS:VERB	-0.340	0.210	[-0.752, 0.072]	-1.618	.106

Supplementary Table S5 Model summary GAMM, smooth terms

B. smooth terms	edf	Ref.df	F-value	p-value
s(Utterance Position)	2.407e+00	2.897	356.377	< 0.0001
s(Utterance Position):TODDLER	4.970e+00	8.000	26.857	< 0.0001
s(Utterance Position):3PLUS	3.136e+00	8.000	6.063	< 0.0001
s(Utterance Position):5PLUS	7.616e-09	8.000	0.000	0.834
s(Utterance Position):ADULTS	2.065e+00	8.000	1.863	< 0.0001

Supplementary Table S6 permutation test results, symmetric Procrustes analysis of cohort pairs, 999 permutations

Pair	SS (sum of squares)	R	p-value
TODDLER:3PLUS	0.5836	0.6453	0.001
3PLUS:5PLUS	0.5878	0.642	0.001
5PLUS:ADULTS	0.6441	0.5966	0.001
TODDLER:ADULTS	0.7262	0.5233	0.001

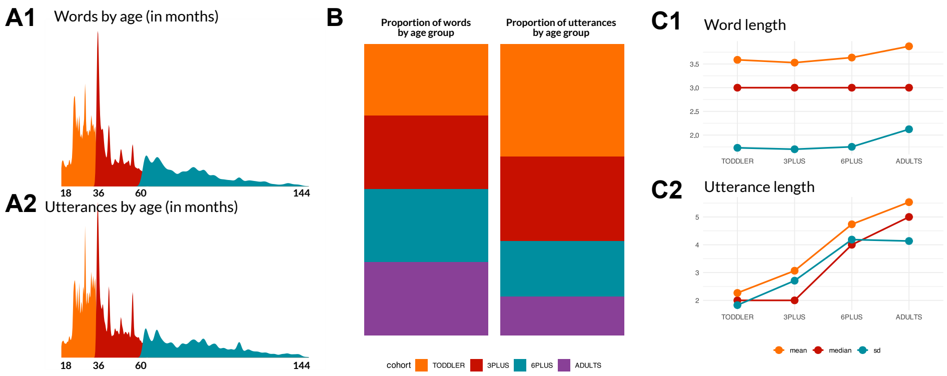

Supplementary Figure S2 Corpus Statistics. Panel A contains density plots of word and sequence counts by child cohort (the adult data is not age labeled). Panel B is a vizualization of proportions of words/sequences from each cohort in the dataset. Panel C shows the development of mean, standard deviation and median for words and sequences across different age groups.

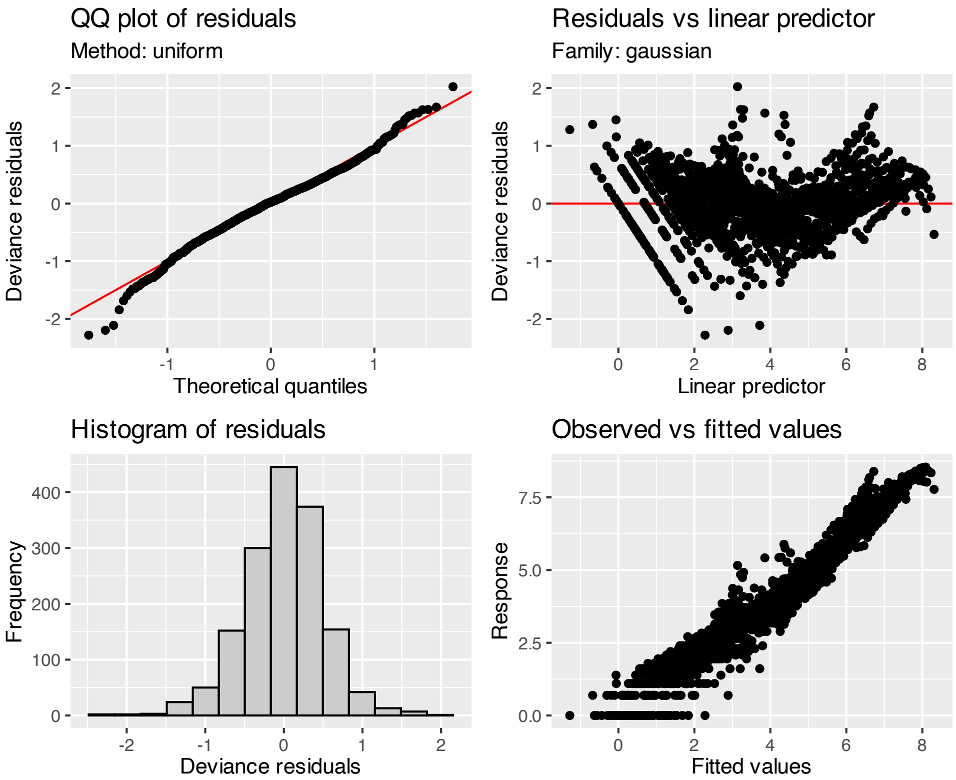

Supplementary Figure S3 Model Diagnostic Plots GAMM

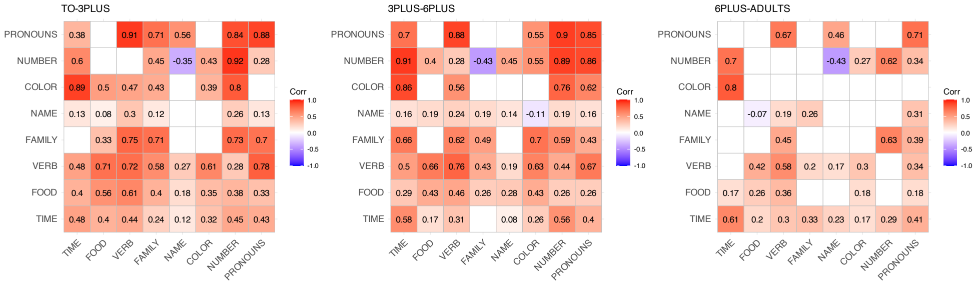

Supplementary Figure S4 Matrix correlation tests, 9999 permutations, r statistic, correlations with p-values > 0.05 are left blank

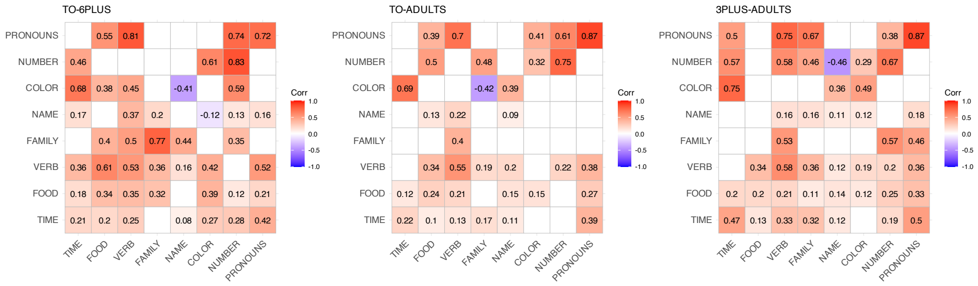

Supplementary Figure S5 Matrix correlation tests, 9999 permutations, r statistic, correlations with p-values > 0.05 are left blank

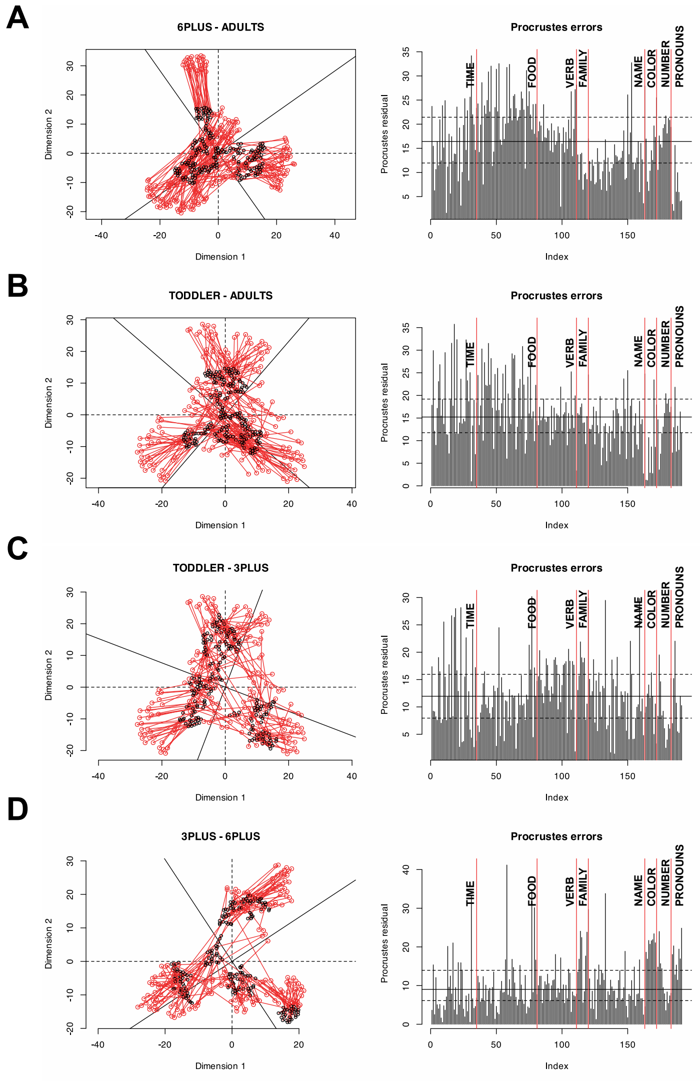

Supplementary Figure S6 Procrustes analyses visualization.

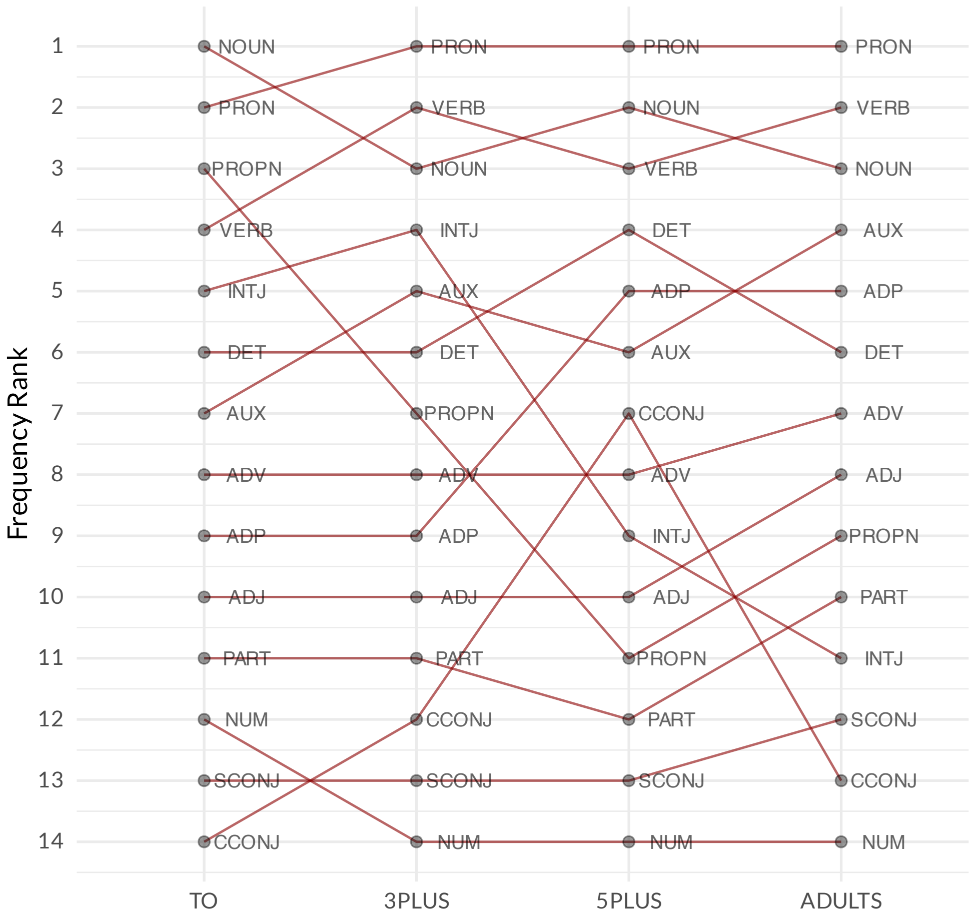

Supplementary Figure S7 The parameters of the parts-of-speech distribution are maintained (see Fig. 4a, main text), and changes in the utterance structure lead to systematic reranking of POS categories.

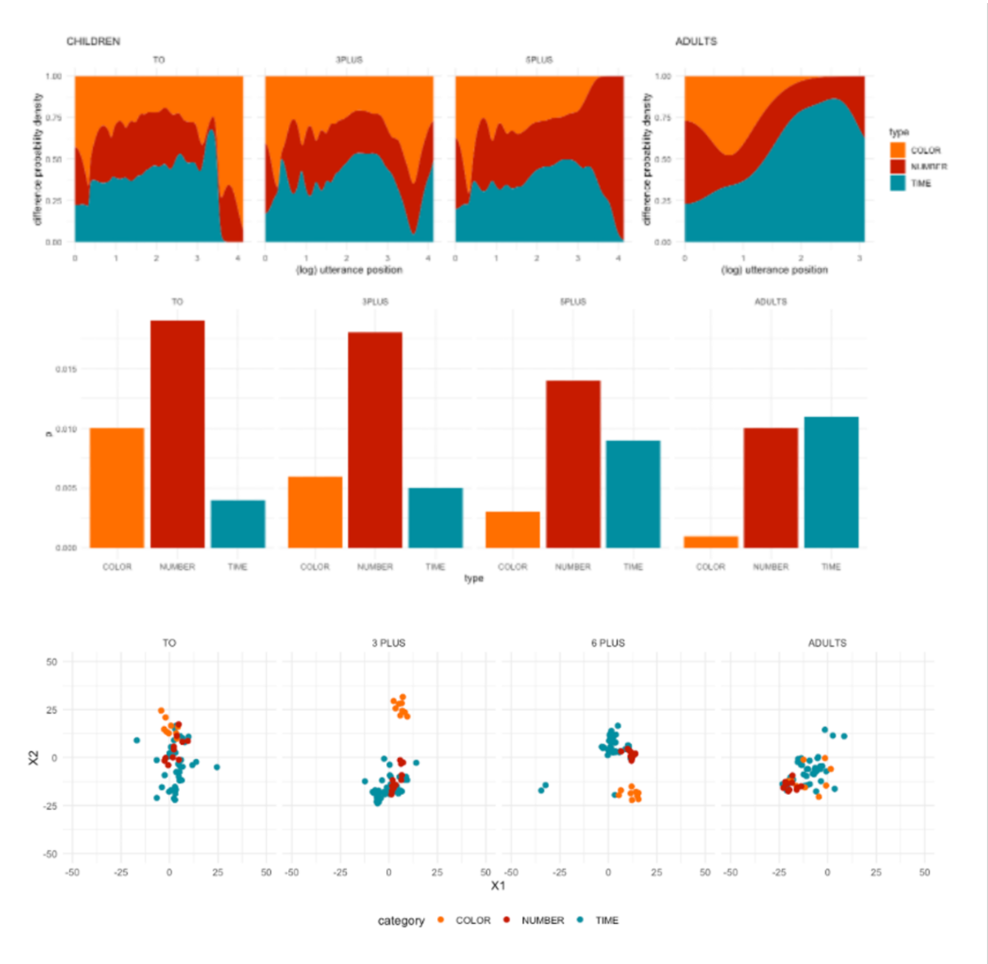

Supplementary Figure S8 Development of conditional density, probability, and cluster structure of color, number, and time words across cohorts.

Supplementary Figure S9 Age × response-time effects on semantic relatedness judgments by trial type. Each panel shows a model-based surface, the tensor-product interaction, for each trial type, shown as partial effects on the log-odds of a correct response (other terms set to zero). The surface height (z-axis) is the log-odds of giving the expected judgment (i.e., "related" for S_H/S_L; "unrelated" for S_U). The y-axis represents age in months; the x-axis represents response time in seconds. Higher surfaces indicate a higher probability of the expected judgment. Two key observations emerge: (i) age and response time both matter but not additively, (ii) at very fast or very slow responses, the age effect reverses across trial types (a cross-over interaction), consistent with different response regimes for highly versus weakly associated word pairs.

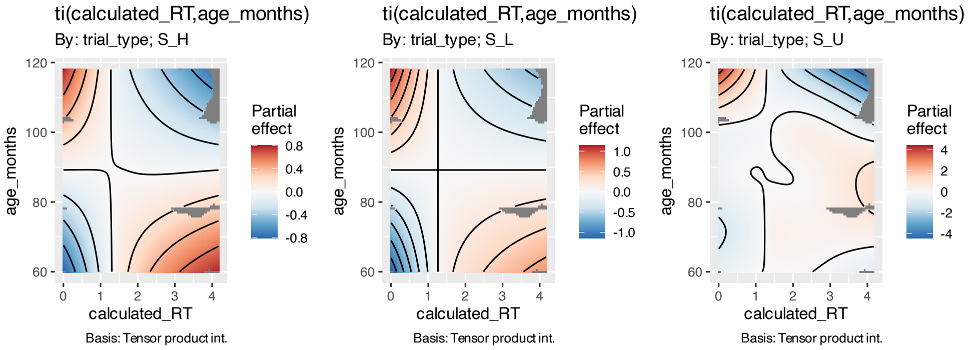

Supplementary Table S7 Parametric (linear) coefficients from generalized additive mixed models (GAMMs) of trial accuracy (binomial, logit link), reported for the baseline model (without cosine similarity) and each cohort-specific embedding (TO, 3PLUS, 5PLUS, ADULTS, MIXED). Entries are log-odds with standard errors (SE), 95% confidence intervals (CIs), Wald z statistics, and two-sided p values. Fixed effects include trial type; participant is modeled with a random intercept (not shown). All models were estimated by fREML.

	model_id	term	Estimate	Std. Error	z value	Pr(>|z|)	95% CI
1	Model_TO	(Intercept)	2.03	0.07	28.01	0.00	[1.89, 2.17]
2	Model_TO	trial_typeS_L	-0.56	0.05	-10.26	0.00	[-0.66, -0.46]
3	Model_TO	trial_typeS_U	-0.78	0.08	-9.57	0.00	[-0.93, -0.63]
4	Model_3PLUS	(Intercept)	1.96	0.08	25.99	0.00	[1.80, 2.12]
5	Model_3PLUS	trial_typeS_L	-0.57	0.05	-10.64	0.00	[-0.67, -0.47]
6	Model_3PLUS	trial_typeS_U	-0.78	0.07	-11.43	0.00	[-0.92, -0.64]
7	Model_5PLUS	(Intercept)	2.03	0.07	28.57	0.00	[1.89, 2.17]
8	Model_5PLUS	trial_typeS_L	-0.55	0.05	-10.42	0.00	[-0.65, -0.45]
9	Model_5PLUS	trial_typeS_U	-0.83	0.07	-11.52	0.00	[-0.97, -0.69]
10	Model_ADULTS	(Intercept)	1.93	0.07	25.94	0.00	[1.79, 2.07]
11	Model_ADULTS	trial_typeS_L	-0.40	0.05	-7.44	0.00	[-0.50, -0.30]
12	Model_ADULTS	trial_typeS_U	-0.81	0.06	-13.64	0.00	[-0.93, -0.69]
13	Model_mixed	(Intercept)	2.00	0.07	28.10	0.00	[1.86, 2.14]
14	Model_mixed	trial_typeS_L	-0.54	0.05	-10.38	0.00	[-0.64, -0.44]
15	Model_mixed	trial_typeS_U	-0.79	0.07	-11.52	0.00	[-0.93, -0.65]
16	Model_baseline	(Intercept)	1.92	0.07	26.59	0.00	[1.78, 2.06]
17	Model_baseline	trial_typeS_L	-0.48	0.05	-10.19	0.00	[-0.58, -0.38]
18	Model_baseline	trial_typeS_U	-0.79	0.05	-17.05	0.00	[-0.89, -0.69]

Supplementary Table S8 Smooth terms and interactions from the same GAMMs. The table reports $s(age\_months)$, $s(calculated\_RT)$, the embedding-specific similarity smooth $s(pair\_dist)$, and the tensor interaction $ti(calculated\_RT,\ age\_months;\ \text{by} = trial\_type)$. For each smooth, we list the effective degrees of freedom (EDF), reference degrees of freedom (ref.df), the test statistic (approximate F or χ^2, as provided by the software), and the two-sided p value. Lower EDF indicates a more constrained (less wiggly) fitted function. Participant random-effect smooths are included for completeness but not interpreted. Models used thin-plate regression splines and were estimated by fREML.

	model_id	smooth	edf	Ref.df	Chi.sq	p-value
1	Model_TO	s(age_months)	2.21	2.58	38.66	0.00
2	Model_TO	s(calculated_RT)	6.18	7.26	265.04	0.00
3	Model_TO	s(pair_dist_TO)	7.54	8.41	67.42	0.00
4	Model_TO	ti(calculated_RT,age_months):trial_typeS_H	4.25	5.68	22.68	0.00
5	Model_TO	ti(calculated_RT,age_months):trial_typeS_L	2.82	3.35	13.92	0.01
6	Model_TO	ti(calculated_RT,age_months):trial_typeS_U	8.08	9.93	72.38	0.00
7	Model_TO	s(participant_id)	184.41	254.00	779.22	0.00
8	Model_3PLUS	s(age_months)	8.25	8.68	76.29	0.00
9	Model_3PLUS	s(calculated_RT)	6.06	7.14	266.31	0.00
10	Model_3PLUS	s(pair_dist_3PLUS)	7.77	8.58	127.37	0.00
11	Model_3PLUS	ti(calculated_RT,age_months):trial_typeS_H	3.95	5.30	22.90	0.00
12	Model_3PLUS	ti(calculated_RT,age_months):trial_typeS_L	2.61	3.14	14.40	0.00
13	Model_3PLUS	ti(calculated_RT,age_months):trial_typeS_U	8.09	9.99	71.68	0.00
14	Model_3PLUS	s(participant_id)	194.30	254.00	957.98	0.00
15	Model_5PLUS	s(age_months)	2.40	2.80	40.27	0.00
16	Model_5PLUS	s(calculated_RT)	6.08	7.17	282.07	0.00
17	Model_5PLUS	s(pair_dist_5PLUS)	7.27	8.26	63.34	0.00
18	Model_5PLUS	ti(calculated_RT,age_months):trial_typeS_H	4.32	5.77	25.48	0.00
19	Model_5PLUS	ti(calculated_RT,age_months):trial_typeS_L	3.00	3.51	18.37	0.00
20	Model_5PLUS	ti(calculated_RT,age_months):trial_typeS_U	7.84	9.66	65.39	0.00
21	Model_5PLUS	s(participant_id)	185.35	254.00	799.29	0.00
22	Model_ADULTS	s(age_months)	8.07	8.57	89.99	0.00
23	Model_ADULTS	s(calculated_RT)	6.13	7.22	287.52	0.00
24	Model_ADULTS	s(pair_dist_ADULTS)	6.80	7.86	50.13	0.00
25	Model_ADULTS	ti(calculated_RT,age_months):trial_typeS_H	3.54	4.83	14.38	0.01
26	Model_ADULTS	ti(calculated_RT,age_months):trial_typeS_L	2.95	3.47	16.40	0.00
27	Model_ADULTS	ti(calculated_RT,age_months):trial_typeS_U	8.38	10.31	67.93	0.00
28	Model_ADULTS	s(participant_id)	191.41	254.00	883.63	0.00
29	Model_mixed	s(age_months)	2.48	2.91	40.43	0.00
30	Model_mixed	s(calculated_RT)	5.90	6.99	282.72	0.00
31	Model_mixed	s(pair_dist)	7.03	8.07	41.91	-0.00
32	Model_mixed	ti(calculated_RT,age_months):trial_typeS_H	4.15	5.51	25.40	0.00
33	Model_mixed	ti(calculated_RT,age_months):trial_typeS_L	2.82	3.35	16.22	0.00
34	Model_mixed	ti(calculated_RT,age_months):trial_typeS_U	8.54	10.35	76.70	0.00
35	Model_mixed	s(participant_id)	186.35	254.00	818.93	0.00
36	Model_baseline	s(age_months)	8.30	8.71	106.45	0.00
37	Model_baseline	s(calculated_RT)	6.64	7.68	290.53	0.00
38	Model_baseline	ti(calculated_RT,age_months):trial_typeS_H	2.08	2.45	13.31	0.00
39	Model_baseline	ti(calculated_RT,age_months):trial_typeS_L	4.70	6.37	12.18	0.06
40	Model_baseline	ti(calculated_RT,age_months):trial_typeS_U	9.40	11.29	89.14	0.00
41	Model_baseline	s(participant_id)	202.14	254.00	1139.08	0.00

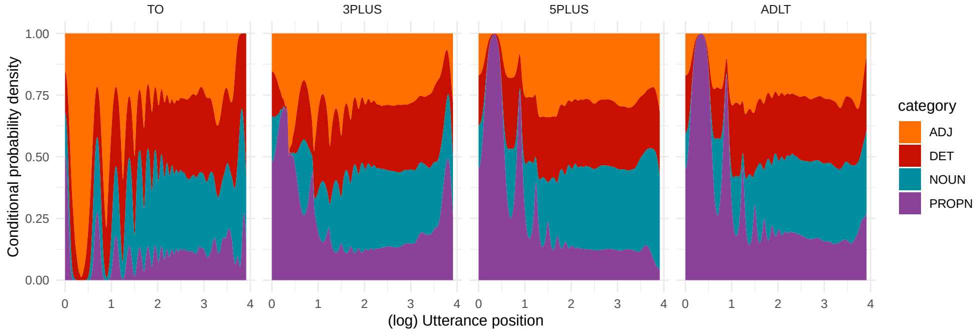

Supplementary Figure S10 Conditional probability density of determiners, adjectives, common and proper nouns in sequences produced by children and adults. The largest differences between cohorts are at the utterance boundaries, where the uncertainty is lowest or highest.

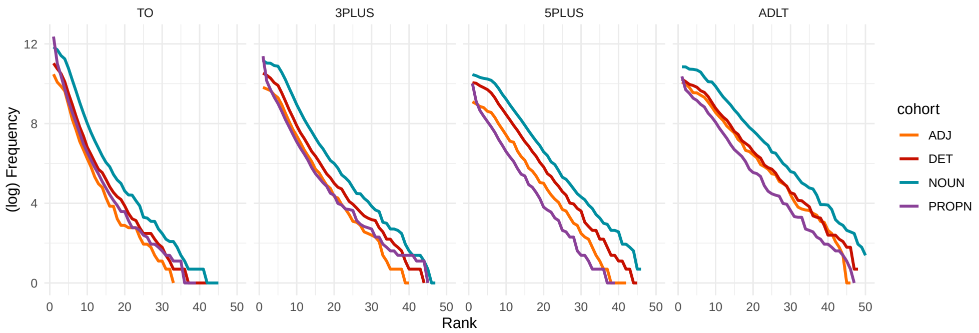

Supplementary Figure S11 Frequency distribution of determiners, adjectives, common and proper nouns at different sequence positions for children and adults.

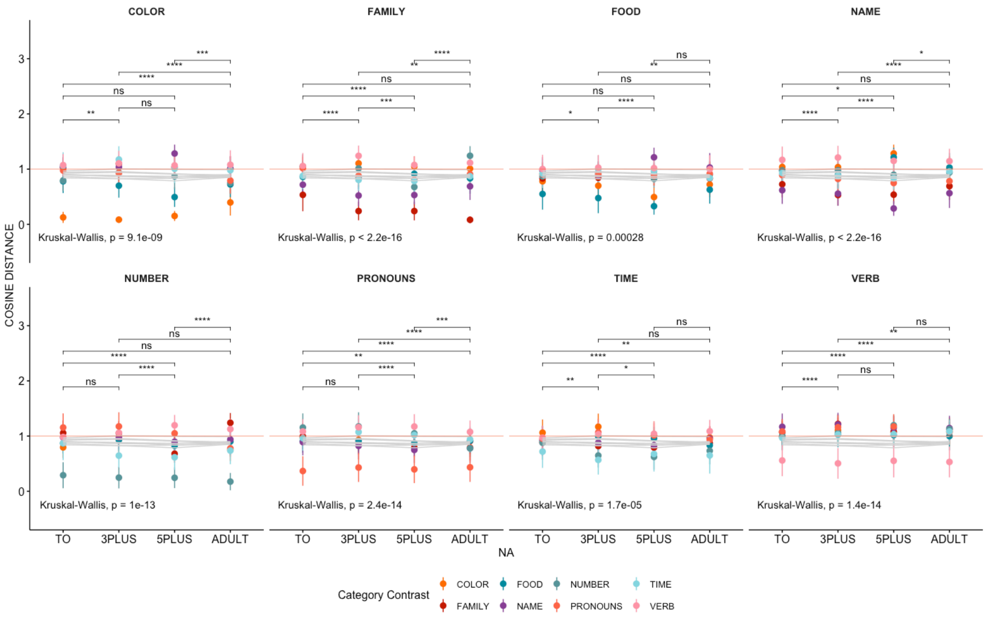

Supplementary Figure S12 Between- and within-category distance as a function of speaker experience. The points represent the mean distance between the source category (panel) and different target categories (color-coded), highlighting differences in the relationship between words from the target category and all other words. The mean distance between categories increases in children and decreases in adults. The degree to which the distance between words within the category (the points with the smallest values at the bottom of each panel) develops in relation to the distance to words from other categories varies with lexical productivity and the grammatical function of words from this category.

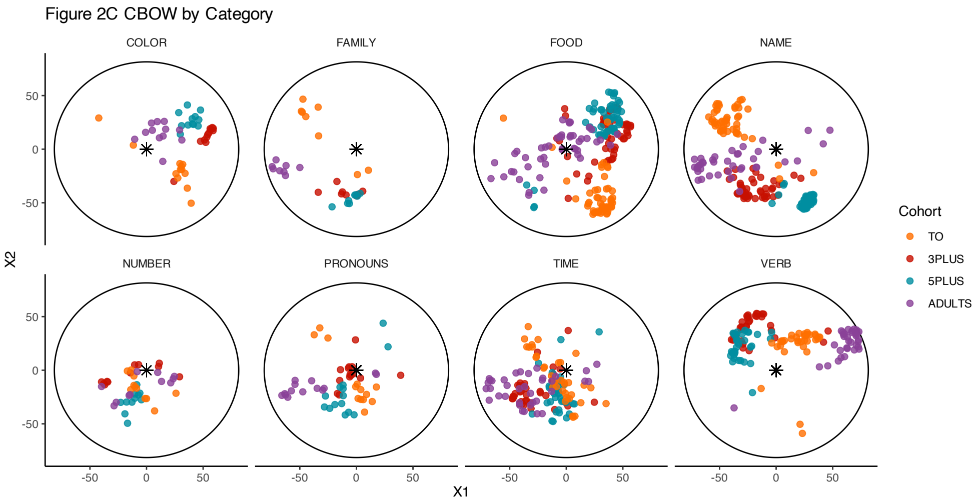

Supplementary Figure S13 Differences between cohorts' embeddings for all eight target categories visualized with t-SNE (circular frame added for reference). Distances between points approximate distances between word vectors; cluster density and distance from the centroid index cohort divergence.

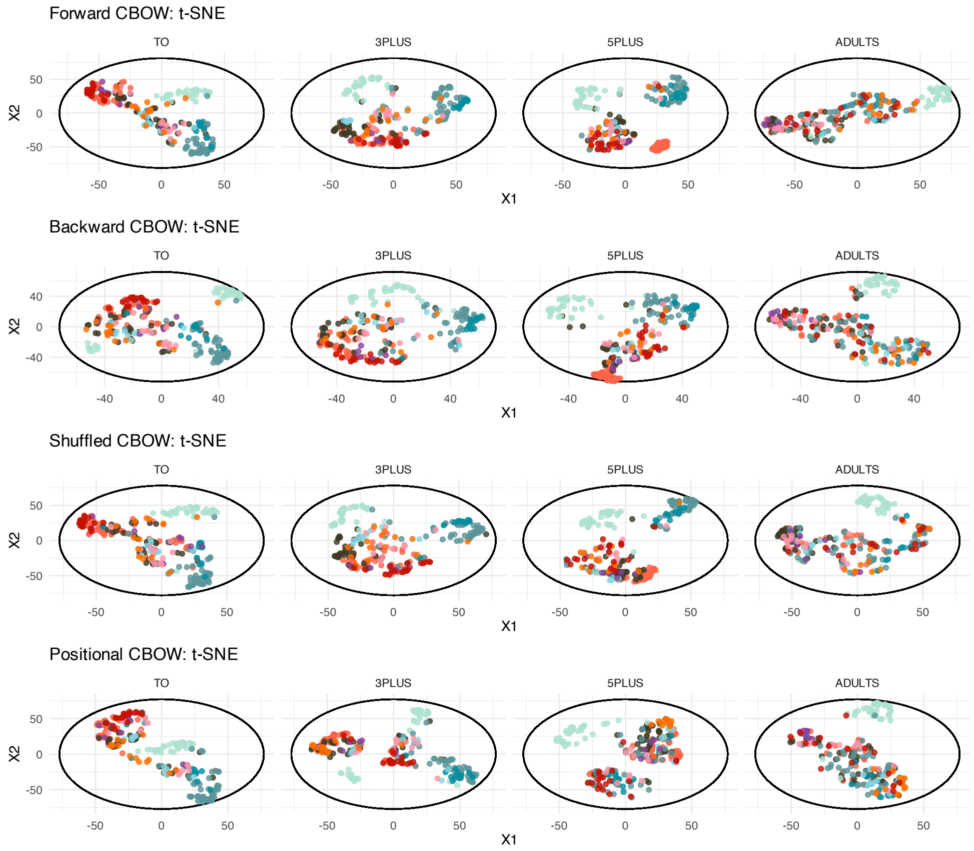

Supplementary Figure S14 t-SNE of CBOW embeddings across cohorts and controls. Columns show cohorts (TO, 3PLUS, 5PLUS, ADULTS); rows show sequence conditions (Forward/original, Backward/reversed, Shuffled, Positional). Points are the common target words, colored by semantic category (NAME, TIME, VERB, FOOD, COLOR, NUMBER, FAMILY, PRONOUNS). Each panel uses a reference ring to keep scale comparable within the facet. Coordinates are 2-D t-SNE projections (perplexity = 20; fixed seed).

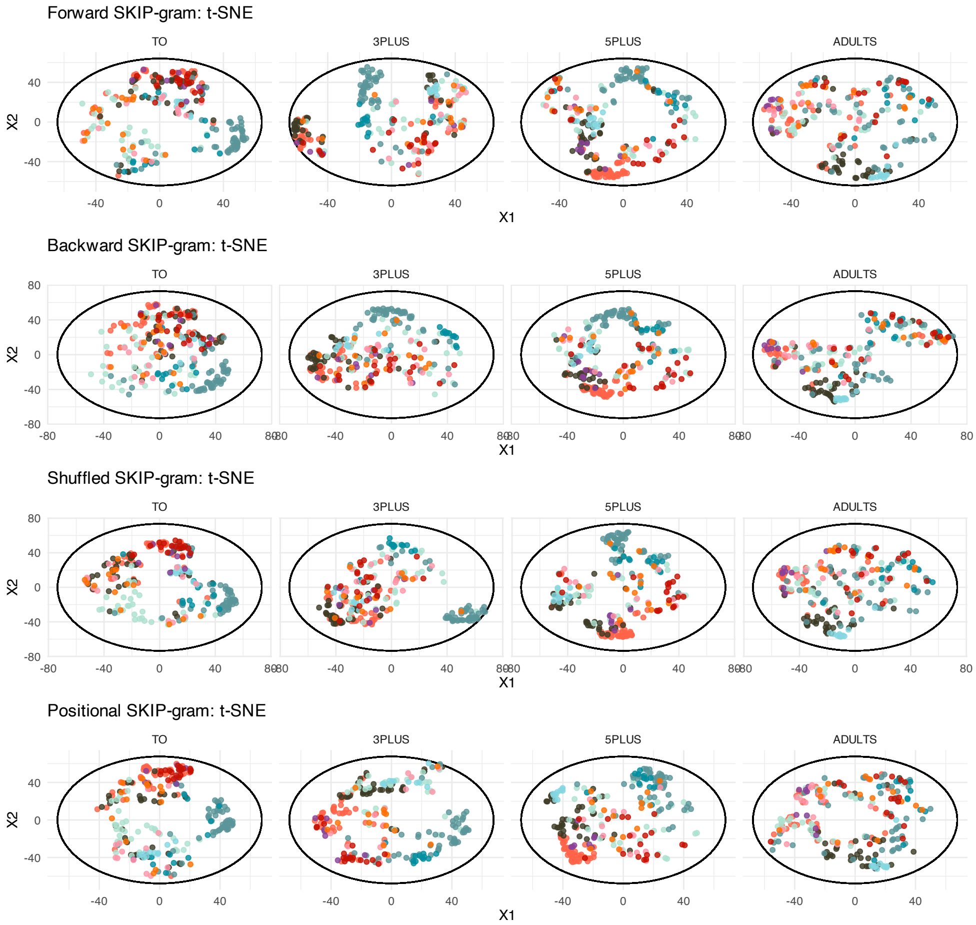

Supplementary Figure S15 t-SNE of Skip-gram embeddings across cohorts and controls. Layout and conventions match the CBOW figure: columns = cohorts; rows = Forward, Backward, Shuffled, Positional sequence conditions; colors denote semantic categories; a reference ring indicates the panel scale. Points are the same common target set, projected with 2-D t-SNE (perplexity = 20; fixed seed).

Supplementary References

[1] Cuesta-Frau, D., Molina-Picó, A., Vargas, B. & González, P. Permutation entropy: Enhancing discriminating power by using relative frequencies vector of ordinal patterns instead of their shannon entropy. *Entropy* **21**, 1013 (2019).

[2] Linke, M. & Ramscar, M. How the probabilistic structure of grammatical context shapes speech. *Entropy* **22**, 90 (2020).
