# Supplementary material for: Sequence structure in children’s speech reveals non-linear development of relations between word categories
Source: Commun Psychol. 2025 Dec 26;4:12. doi: 10.1038/s44271-025-00380-w (PMC12847997; doi:10.1038/s44271-025-00380-w)
Supplement: Supplementary file 3 — Reporting Summary [file 44271_2025_380_MOESM3_ESM.pdf]

nature portfolio

Corresponding author(s): Maja Linke

Last updated by author(s): Apr 15, 2025

Reporting Summary

Nature Portfolio wishes to improve the reproducibility of the work that we publish. This form provides structure for consistency and transparency in reporting. For further information on Nature Portfolio policies, see our Editorial Policies and the Editorial Policy Checklist.

Statistics

For all statistical analyses, confirm that the following items are present in the figure legend, table legend, main text, or Methods section.

n/a	Confirmed	
☐	☒	The exact sample size (*n*) for each experimental group/condition, given as a discrete number and unit of measurement
☐	☒	A statement on whether measurements were taken from distinct samples or whether the same sample was measured repeatedly
☐	☒	The statistical test(s) used AND whether they are one- or two-sided<br>*Only common tests should be described solely by name; describe more complex techniques in the Methods section.*
☐	☒	A description of all covariates tested
☐	☒	A description of any assumptions or corrections, such as tests of normality and adjustment for multiple comparisons
☐	☒	A full description of the statistical parameters including central tendency (e.g. means) or other basic estimates (e.g. regression coefficient) AND variation (e.g. standard deviation) or associated estimates of uncertainty (e.g. confidence intervals)
☐	☒	For null hypothesis testing, the test statistic (e.g. *F*, *t*, *r*) with confidence intervals, effect sizes, degrees of freedom and *P* value noted<br>*Give P values as exact values whenever suitable.*
☒	☐	For Bayesian analysis, information on the choice of priors and Markov chain Monte Carlo settings
☐	☒	For hierarchical and complex designs, identification of the appropriate level for tests and full reporting of outcomes
☐	☒	Estimates of effect sizes (e.g. Cohen's *d*, Pearson's *r*), indicating how they were calculated

Our web collection on statistics for biologists contains articles on many of the points above.

Software and code

Policy information about availability of computer code

Data collection	All data used in this study are publicly available. The CHILDES database was accessed via the childesr API (version 2021.1) for R, and the subtitle datasets were accessed via the subs2vec API (Python) from GitHub.
Data analysis	Analyses were conducted using R (version 4.4.2) and Python. Child language data were accessed via the childesr API (version 2021.1) for R, which interfaces with the CHILDES database. Adult datasets were obtained using the subs2vec Python API, available at https://github.com/jvparidon/subs2vec. All custom code used for data access and analysis is available on GitHub and in an OSF repository: https://osf.io/qxb9j/.<br><br>Part-of-speech tagging was performed using the spaCyR package (version 1.3.0) in R, with the underlying Python spaCy library (version 3.7.2) and the en_core_web_sm model.<br><br>R packages used for data preprocessing and analyses include: tidyverse (version 2.0.0), fitdistrplus (version 1.2.1), word2vec (version 0.4.0), tsne (version 0.1.3.1), udpipe (version 0.8.11), StatMatch (version 1.4.2), mgcv (version 1.9.1), mgcViz (version 0.2.0), gratia (version 0.9.2), philentropy (version 0.8.0) and vegan (version 2.6.8).

For manuscripts utilizing custom algorithms or software that are central to the research but not yet described in published literature, software must be made available to editors and reviewers. We strongly encourage code deposition in a community repository (e.g. GitHub). See the Nature Portfolio guidelines for submitting code & software for further information.

Data

Policy information about availability of data

All manuscripts must include a data availability statement. This statement should provide the following information, where applicable:

- Accession codes, unique identifiers, or web links for publicly available datasets
- A description of any restrictions on data availability
- For clinical datasets or third party data, please ensure that the statement adheres to our policy

OpenNeuro dataset ds003604

The dataset comprises N = 322 children assessed longitudinally at 5.5–6.5, 7–8, and 8.5–10 years, with behavioral logs for tasks probing phonological, semantic, plausibility, and grammatical processing. The resource is BIDS-formatted and distributed on OpenNeuro under a CC0 license; the accompanying Data Descriptor provides methodological detail. For this work, we used only the behavioral logs (*_events.tsv) and task-level Stimulus Characteristics tables together with participants.tsv. Data were accessed via the OpenNeuro Python API (openneuro-py), accession ds003604 (version 1.0.2), on 15 June 2025. Key references: Scientific Data article (Wang et al., 2022) and the OpenNeuro dataset page., doi 10.18112/openneuro.ds003604.v1.0.2

Wang, J., Lytle, M. N., Weiss, Y., Yamasaki, B. L., & Booth, J. R. (2022). A longitudinal neuroimaging dataset on language processing in children ages 5, 7, and 9 years old. Scientific Data, 9(1), 4.

Analysis was performed in Python (3.10) using the OpenNeuro Python client (openneuro-py), PyBIDS, pandas, numpy and tqdm. All scripts to (i) fetch ds003604 via the OpenNeuro API and (ii) assemble the behavioural table (merging *_events.tsv, participants.tsv, and task-specific *_Stimulus_Characteristics.tsv) are available in our repository.

The CHILDES database comprises a diverse set of child language transcripts collected across multiple studies over several decades. Data were originally collected by contributing researchers using naturalistic and experimental methods, and are curated and maintained by the TalkBank project to support open access and standardized formats for language acquisition research.

The subs2vec repository offers subtitle data and pre-trained word embeddings for 55 languages, derived from the OpenSubtitles corpus. The data sets contain utterances from from naturalistic, conversational language found in subtitles of movies and television shows. For more details and access to the code and models, visit the GitHub repository: https://github.com/jvparidon/subs2vec.

Child language data were accessed via the childesr API (version 2021.1) for R, which interfaces with the CHILDES database. Adult datasets were obtained using the subs2vec Python API, available at https://github.com/jvparidon/subs2vec. All custom code used for data access is available on GitHub and in an OSF repository: https://osf.io/qxb9j/.

Research involving human participants, their data, or biological material

Policy information about studies with human participants or human data. See also policy information about sex, gender (identity/presentation), and sexual orientation and race, ethnicity and racism.

Reporting on sex and gender

NA

Reporting on race, ethnicity, or other socially relevant groupings

NA

Population characteristics

OpenNeuro dataset ds003604
The dataset comprises N = 322 children assessed longitudinally at 5.5–6.5, 7–8, and 8.5–10 years

CHILDES Database

The child language data were drawn from selected corpora in the CHILDES database, a large, publicly available repository of transcripts documenting child–caregiver interactions. The dataset includes children aged 18 months to 12 years, sampled across three age groups (18 months–3 years, 3–5 years, and 5–12 years). The corpora represent a mix of monolingual and bilingual children, though only English-language transcripts were used in this study. Information on sex, socioeconomic status, and geographic background varies across corpora and was not used as a sampling criterion. As a result, the sample is not demographically representative of a specific population but reflects a diverse range of naturalistic child language environments collected across different studies and countries over several decades.

Recruitment

NA

Ethics oversight

OpenNeuro dataset ds003604

Data are public, de-identified secondary data from the UT Austin IRB-approved study (parental consent / child assent obtained by the original team). Our reuse required no additional ethics review.

CHILDES Database

CHILDES/TalkBank provides public, de-identified transcripts contributed by independent research groups. Original data collection for each contributing corpus was carried out under the investigators' institutional approvals with informed consent/assent as applicable. Our secondary analysis of these de-identified data did not require additional ethics approval.

Note that full information on the approval of the study protocol must also be provided in the manuscript.

Field-specific reporting

Please select the one below that is the best fit for your research. If you are not sure, read the appropriate sections before making your selection.

☐ Life sciences ☒ Behavioural & social sciences ☐ Ecological, evolutionary & environmental sciences

For a reference copy of the document with all sections, see nature.com/documents/nr-reporting-summary-flat.pdf

Behavioural & social sciences study design

All studies must disclose on these points even when the disclosure is negative.

Study description	This study is a quantitative corpus analysis based on existing language data. We analyzed naturalistic language use in child and adult corpora using computational and statistical methods. No experimental manipulation or qualitative coding was involved.
Research sample	The child data were sampled from transcripts in the CHILDES database, a publicly available collection of child language corpora contributed by researchers over several decades. The sample includes children aged 18 months to 12 years, stratified into three age groups (18 months–3 years, 3–5 years, and 5–12 years), with approximately equal numbers of samples per group and an even distribution of ages within each. Sex was not used as a criterion for sampling. Due to the diverse origins of the CHILDES corpus, the sample is not fully representative of any single population.<br><br>The adult data were sampled at random from the subtitle corpus used in the subs2vec repository, which consists of English-language subtitles from movies and television shows. This corpus reflects naturalistic, conversational language but is not demographically annotated and should not be considered representative of any specific adult population.
Sampling strategy	CHILDES -- Participants were sampled using a stratified sampling approach based on age. The dataset was divided into three age groups: 18 months to 3 years, 3 to 5 years, and 5 to 12 years. To ensure balanced representation across groups, we subsampled to obtain similar sample sizes per group. Within each age group, samples were further filtered to reduce collection bias and maintain an even distribution of ages. For the adult data, samples were drawn at random from the subtitle corpus used in the subs2vec repository.
Data collection	The OpenNeuro dataset (accession ds003604) comprises longitudinal lab sessions in which children completed structured, speeded auditory judgment tasks. On each trial, stimuli were presented over headphones and the child provided a binary response with a button press; reaction time (RT) and accuracy were logged by the task software. Trial timing and responses were archived in BIDS-compatible *_events.tsv files (e.g., onset, duration, trial_type, stim_file, response, RT), and task-level stimulus descriptors (e.g., wordA, wordB, total_stim_duration, pause_duration) were provided in the accompanying *_Stimulus_Characteristics.tsv sheets. For the present work, we accessed the dataset programmatically via the OpenNeuro Python API and used only the behavioral components from the semantic relatedness (S_) and phonetic/sound discrimination (P_) tasks; imaging data were not analyzed. We merged trial logs with stimulus descriptors and participant metadata (participants.tsv) to obtain a single trial-level table for analysis.<br><br>The data analyzed in this study were drawn from existing corpora. Child language data were obtained from the CHILDES database, which aggregates transcripts of child–caregiver interactions collected by contributing researchers across a range of naturalistic and experimental settings. Audio or video recordings were originally made using standard recording equipment (e.g., microphones, camcorders) and later transcribed following the CHAT transcription conventions, a standardized format developed for TalkBank.<br><br>Adult language data were sourced from the subs2vec repository, which is based on subtitle files from movies and television shows in the OpenSubtitles corpus. These subtitles were originally created for broadcast and home media distribution, and reflect informal, conversational adult speech. No new data were collected as part of this study.
Timing	No new data were collected for this study. The child language data were drawn from corpora in the CHILDES database, which includes transcripts collected between the 1960s and early 2010s, depending on the contributing study. The adult subtitle data, sourced from the subs2vec repository based on the OpenSubtitles corpus, reflect dialogues from movies and television shows released between approximately the 1980s and 2018.
Data exclusions	To ensure comparability across age groups and reduce collection bias, we included only a curated subset of 75 CHILDES corpora. The full list of collections used in these analyses is provided in the Supplementary Materials.<br><br>For the OpenNeuro dataset (ds003604) we restricted analyses to the behavioral logs from the semantic relatedness (S_) and sound/phonetic discrimination (P_) tasks. The plausibility (SP_) and grammaticality (G_) tasks, as well as all imaging data and non-behavioral files, were excluded a priori. Within the selected tasks, we retained all trials with a recorded button response and reaction time; rows lacking either value were dropped.
Non-participation	NA

Randomization | NA

Reporting for specific materials, systems and methods

We require information from authors about some types of materials, experimental systems and methods used in many studies. Here, indicate whether each material, system or method listed is relevant to your study. If you are not sure if a list item applies to your research, read the appropriate section before selecting a response.

Materials & experimental systems

n/a	Involved in the study
☒	☐ Antibodies
☒	☐ Eukaryotic cell lines
☒	☐ Palaeontology and archaeology
☒	☐ Animals and other organisms
☒	☐ Clinical data
☒	☐ Dual use research of concern
☒	☐ Plants

Methods

n/a	Involved in the study
☒	☐ ChIP-seq
☒	☐ Flow cytometry
☒	☐ MRI-based neuroimaging

Plants

Seed stocks

Report on the source of all seed stocks or other plant material used. If applicable, state the seed stock centre and catalogue number. If plant specimens were collected from the field, describe the collection location, date and sampling procedures.

Novel plant genotypes

Describe the methods by which all novel plant genotypes were produced. This includes those generated by transgenic approaches, gene editing, chemical/radiation-based mutagenesis and hybridization. For transgenic lines, describe the transformation method, the number of independent lines analyzed and the generation upon which experiments were performed. For gene-edited lines, describe the editor used, the endogenous sequence targeted for editing, the targeting guide RNA sequence (if applicable) and how the editor was applied.

Authentication

Describe any authentication procedures for each seed stock used or novel genotype generated. Describe any experiments used to assess the effect of a mutation and, where applicable, how potential secondary effects (e.g. second site T-DNA insertions, mosiacism, off-target gene editing) were examined.
